# Quantitative Proteomics Analysis of Membrane Proteins in *Enterococcus faecalis* With Low-Level Linezolid-Resistance

**DOI:** 10.3389/fmicb.2018.01698

**Published:** 2018-07-27

**Authors:** Jia Yan, Yun Xia, Mi Yang, Jiaqi Zou, Yingzhu Chen, Dawei Zhang, Liang Ma

**Affiliations:** ^1^Department of Clinical Laboratory, The First Affiliated Hospital of Chongqing Medical University, Chongqing, China; ^2^Critical Care Medicine Department, Clinical Center, National Institutes of Health, Bethesda, MD, United States

**Keywords:** *Enterococcus faecalis*, linezolid, low-level resistance, membrane proteins, quantitative proteomics

## Abstract

Despite increasing reports of low-level linezolid-resistant enterococci worldwide, the mechanism of this resistance remains poorly understood. Previous transcriptome studies of low-level linezolid-resistant *Enterococcus faecalis* isolates have demonstrated a number of significantly up-regulated genes potentially involved in mediation of drug resistance. However, whether the transcriptome faithfully reflects the proteome remains unknown. In this study, we performed quantitative proteomics analysis of membrane proteins in an *E. faecalis* isolate (P10748) with low-level linezolid-resistance in comparison with two linezolid-susceptible strains 3138 and ATCC 29212, all of which have been previously investigated by whole transcriptome analysis. A total of 8,197 peptides associated with 1,170 proteins were identified in all three isolates with false discovery rate (FDR) at 1% and *P* < 0.05. There were 14 significantly up-regulated and 6 significantly down-regulated proteins in strain P10748 compared to strains 3138 and ATCC 29212, which were in general positively correlated with transcription levels revealed in previous transcriptome studies. Our analysis suggests that the low-level linezolid-resistance in *E. faecalis* is conferred primarily by the ATP-binding cassette protein OptrA through ribosomal protection and, possibly, also by the enterococcal surface protein (Esp) and other proteins through biofilm formation. The genetic transfer of *optrA* is potentially regulated by the surface exclusion protein Sea1, conjugal transfer protein TraB, replication protein RepA and XRE family transcription regulator protein. This report represents the first investigation of the mechanisms of linezolid-resistance in *E. faecalis* by a quantitative proteomics approach.

## Introduction

*Enterococcus faecalis* is a common Gram-positive opportunistic bacterium ubiquitously inhabiting the gastrointestinal tract of humans and animals (Mundy et al., 2000; Yuen and Ausubel, 2014). In people with weakened immunity, *E. faecalis* can spread to other systems causing life-threatening infections, including septicemia, endocarditis, meningitis, and urinary tract infections. Over the past few decades, *E. faecalis* has emerged as one of the leading causes of hospital-acquired infection. Treatment of *E. faecalis* infection is challenging due to its resistance to many commonly used antimicrobial agents including vancomycin. One of the most widely prescribed antibiotics for vancomycin-resistant enterococci is linezolid, a completely synthetic drug approved for clinical use since 2000 (Diekema and Jones, 2001; Moellering, 2003; Mendes et al., 2014).

Although initially thought to have a low probability of developing drug resistance due to its unique mechanism of antibacterial action (Meka and Gold, 2004), apparent resistance to linezolid has been increasingly reported in *E. faecalis* clinical isolates worldwide (Gonzales et al., 2001; Cai et al., 2015; Cui et al., 2016; Pfaller et al., 2017). It has been found that clinical resistance to linezolid can be mediated by point mutations in the chromosomal 23S rRNA gene or protein-coding genes *rplC* and *rplD* that encode 50S ribosomal proteins L3 and L4, respectively, and by plasmid-encoded ribosomal methylase Cfr or ATP-binding cassette (ABC) protein OptrA (Miller et al., 2014; Wang et al., 2015). Recently, it has been demonstrated that OptrA belongs to the ABC-F subfamily that functions to mediate antibiotic resistance through ribosomal protection (Sharkey et al., 2016; Murina et al., 2018; Sharkey and O’Neill, 2018). In addition to high level, clinically relevant resistance, which has been tentatively defined as an increase in the minimum inhibitory concentration (MIC) of >64 mg/L (Cho et al., 2017), low-level resistance to linezolid in *E. faecalis* has gained growing attention over the past decade (Jones et al., 2009; Ross et al., 2011). In general for all bacteria, low-level antibacterial resistance has been defined as an increase in MIC above that of the average susceptible bacterial population but below the threshold for clinically relevant resistance (Baquero, 2001). Low-level antibacterial resistance has risen as an important issue since it may act as a stepping-stone to higher levels of resistance. Understanding the mechanisms of low-level resistance is essential to prevent and control bacterial infections and to predict the emergence of resistance to a new antibiotic before its clinical onset (Martinez et al., 2007). Currently, there is no consensus definition for low-level linezolid-resistance in *Enterococcus* spp., while an MIC range of 8–16 mg/L (Cho et al., 2017) has been suggested. Considering this report as well as the breakpoints of MICs recommended by the European Committee on Antimicrobial Susceptibility Testing (version 8.1, 2018) and the U.S. Clinical and Laboratory Standards Institute Performance Standards for Antimicrobial Susceptibility Testing (28th Edn, 2018), we tentatively define an MIC range of 8–16 mg/L for the low-level linezolid-resistance in *E. faecalis* in the current manuscript.

We have previously reported the presence of low-level linezolid-resistance in *E. faecalis* clinical isolates without mutations in 23S rRNA, *rplC* or *rplD*, and without a *cfr*-bearing plasmid (Wang et al., 2014). Whole transcriptome analysis revealed significant up-regulation of several genes in an *E. faecalis* clinical isolate with low-level linezolid-resistance in comparison with linezolid-susceptible strains (Hua et al., 2018). These genes include *esp*, *optrA*, and *fexA*, which encode enterococcal surface protein (Esp), ABC-F protein (OptrA), and major facilitator superfamily transporter (FexA), respectively. We hypothesized that dramatic upregulation of their transcription may mirror their roles in low-level resistance to linezolid in *E. faecalis.* However, an upregulated transcription does not necessarily correlate with a higher translation. As has been reported from studies of various organisms, genome-wide correlation between mRNA and protein expressions is notoriously poor, fluctuating around 40% explanatory power among numerous studies (Vogel and Marcotte, 2012). In addition, there has been no report of proteomic analysis of linezolid-resistance in *E. faecalis.*

Over the past decade, quantitative proteomics has emerged as a powerful tool in biological research (Li et al., 2017), including antibiotic resistance in some bacteria (Otto et al., 2014; Tiwari and Tiwari, 2014). This development prompted us to undertake a proof-of-concept study to test the feasibility of this approach for profiling differentially expressed proteins in the membrane of the same set of *E. faecalis* isolates that have been previously examined by whole transcriptome analysis, including the strain P10748 with low-level linezolid-resistance and two linezolid-susceptible strains 3138 and ATCC 29212 (Hua et al., 2018). We employed a quantitative proteomics approach that relies on tandem mass tags (TMT)-labeling and nano-liquid chromatography-tandem mass spectrometry (nano LC–MS/MS).

## Materials and Methods

### Bacterial Strains and Susceptibility Testing

*Enterococcus faecalis* strains P10748 and 3138 were isolated from necrotic tissues and secretions in two infected patients as described in our early studies (Wang et al., 2014; Hua et al., 2018). The *E. faecalis* ATCC 29212 strain was obtained from American Type Culture Collection and used as the standard quality control strain. MICs were determined to be 8 mg/L for strain P10748, 2 mg/L for strain 3138, and 2 mg/L for ATCC 29212 as described previously (Hua et al., 2018). The former strain was designated as the low-level linezolid-resistant strain, and the latter two strains were susceptible strains.

### Membrane Protein Extraction

All *E. faecalis* isolates were cultured using the same conditions as previously described for transcriptome analysis (Hua et al., 2018). Bacterial cells were harvested from culture grown to mid-exponential phase, resuspended in 20 mL of ice cold Tris-HCl (10 mM, pH 7.4) and lysed by ultrasonication in ice bath (at 20% power capacity, and 110 cycles of power on for 5 s and off for 6 s). The cell lysate was mixed with 500 μL bacterial protease inhibitor cocktail (Sigma) to suppress endogenous proteinase activity, and 1,000 U DNase and 500 U RNase (Sigma) in the presence of 10 mM MgCl_2_ to degrade nucleic acids. After incubating on ice for 1 h, the supernatant was collected by centrifugation at 3,000 *g* for 10 min at 4°C and stored at -20°C overnight.

The protein lysate was thawed and ultra-centrifugated at 100,000 *g* for 1 h at 4°C. The pellet was collected and homogenized in 10 mL of 2% Sodium *N*-Lauroyl Sarcosinate and 10 mM Tris-HCl, pH 7.4, for 30 min on ice. The resulting solution was ultra-centrifugated at 100,000 *g* for 1 h at 4°C. The pellet was subjected to a second round of homogenization and centrifugation under the same conditions. The final protein preparation was quantitated using Bradford assay, and its integrality was checked using SDS-PAGE. Protein samples were sent to the Beijing Genomics Institute (BGI) (Shenzhen, China) for further processing as described below.

### Peptide Digestion and TMT Labeling

Peptide digestion was performed following the previously reported protocol (Wen et al., 2014; Zhu et al., 2016). Briefly, 100 μg of each membrane protein sample was subjected to sequential treatment including denaturation with five times the volume of acetone, reduction with 10 mM dithiothreitol, and alkylation with 55 mM iodoacetamide. The resulting mixture was first digested with 2.5 μg of trypsin at 37°C for 4 h, after that the same amount of trypsin was added again to the mixture and incubated for an additional 8 h. The protein digests were desalted using a Strata-X column (Phenomenex, Torrance, CA, United States) and dried in the SpeedVac. The precipitate was dissolved in 0.1 M triethylamine borane (TEAB) to a final concentration of 3.74 μg/μL and then mixed at room temperature for 2 h with the following TMT isotopes: 126, 127N, and 127C for ATCC 29212; 128N, 128C, and 129N for strain P10748; and 129C, 130C, and 130N for strain 3138. The labeled peptides were pooled together for Nano LC–MS/MS analysis (**Supplementary Figure S1**).

### Nano LC–MS/MS Analysis of Labeled Peptides

Pooled peptides were dried in a SpeedVac and then dissolved in 2 mL of Buffer A (5% acetonitrile, pH 9.8). Fractionation was carried out in a HPLC system (LC-20AB, Shimadzu, Kyoto, Japan) using Gemini C18 column (5 μm, 250 mm × 4.6 mm, Phenomenex, Torrance, CA, United States). A total of 20 fractions were collected at a rate of 1 mL/min with Buffer B (95% acetonitrile, pH 9.8) with a multi-step gradual increase in the Buffer B concentration as follows: 0% for 3 min, 5% for 10 min, 5–35% for 40 min, and 35–95% for 1 min.

The fractions were freeze-dried, re-dissolved in Solvent A (2% acetonitrile, 0.1% formic acid) and centrifuged at 20,000 *g* for 10 min. The supernatant was desalted and loaded onto the Prominence Nano HPLC system (LC-20AD, Shimadzu, Kyoto, Japan) equipped with a C18 column (ID 75 μm, 3 μm particles, 15 cm). Separation was run at a rate of 300 nL/min with Solvent B (98% acetonitrile, 0.1% formic acid) with a multi-step gradual change of the Solvent B concentration as follows: 5% for 0–8 min, 8–35% for 8–43 min, 35–60% for 43–48 min, 60–80% for 48–50 min, 80% for 50–55 min, and 5% for 55–65 min.

Separated peptides were further analyzed using the Q-Exactive mass spectrometer (Thermo Fisher Scientific, San Jose, CA, United States) with its parameters set as a data-dependent acquisition mode with a full MS scan from 350 to 1600 *m/z* at a resolution of 70,000, a full MS2 scan from 100 *m/z* at a resolution of 17,500, charge state screening parameters at 2+ to 7+, and dynamic exclusion setting of 15 s. Each isolate was assessed in triplicate.

### Spectrum Data Analysis

Spectrum data analysis was carried out using the pipeline previously established at BGI (Wen et al., 2014; Zhu et al., 2016). Briefly, raw spectra data were processed with Proteome Discover 1.2 (Thermo Fisher Scientific, Waltham, MA, United States) and searched with MASCOT software 2.3.02 (Matrix Science, London, United Kingdom) against the Enterococcus transcriptome database. In the database search, the following options were used: type of search = MS/MS Ion search; peptide mass tolerance = 20 ppm; fragment mass tolerance = 0.05 Da; enzyme = trypsin; mass values = monoisotopic; fixed modification: carbamidomethyl (C), TMT10plex (K), TMT10plex (N-term); variable modification: oxidation (M), TMT10plex (Y). At least one unique peptide was required for protein identification and quantification. The false discovery rate (FDR) at 1% was set in both PSM (peptide spectrum match)-level and protein-level using a Mascot Percolator algorithm (Brosch et al., 2009) by IQuant software (Wen et al., 2014) with the picked protein FDR strategy (Savitski et al., 2015).

All identified proteins were searched against the Gene Ontology (GO), Cluster of Orthologous Groups (COG), and KEGG databases for function and pathway annotation. GO and pathway enrichment analyses were performed to explore the biological functions and enriched pathways of differentially expressed proteins between compared samples.

### Verification of the Surface Exclusion Protein-Encoding Gene *sea1* and Its Transcription

Genomic DNA was extracted from *E. faecalis* culture using the HiPure Bacterial DNA Kit (Magen, Guangzhou, China) according to the manufacturer’s instruction. The *sea1* gene was detected by polymerase chain reaction (PCR) using primers 5′-CAGGCAGCAGAACAAGCG-3′ and 5′-GTCGCTTTGCTTCTCGTTCA-3′ and the following thermocycling conditions: 94°C for 5 min, followed by 35 cycles of 94°C for 30 s, 56°C for 30 s, and 72°C for 60 s. PCR products were purified using the Spin Column DNA Gel Extraction Kit (BBI Life Science, Shanghai, China) following the manufacturer’s instruction. Purified PCR products were directly sequenced commercially in both directions. The *sea1* gene sequence obtained from strain P10748 was deposited into GenBank (GenBank Accession No. MH510241).

Total RNA was extracted from *E. faecalis* culture using the Trizol reagent (Life Technologies). cDNA was synthesized from total RNA using the PrimeScript^TM^ RT reagent kit with gDNA Eraser and RT Primer Mix, which contains both the oligo (dT) and random hexomers (Takara, Japan). A pair of primers was designed to amplify the *E. faecalis seal* gene, including forward primer, 5′-AAGCAGTCGCAGACCAACA-3′ and reverse primer, 5′-TCCATAACCCATTCTTCCATC-3′. Reverse-transcription PCR (RT-PCR) was performed immediately after cDNA synthesis. The thermocycling conditions were as follows: 94°C for 5 min, followed by 35 cycles of 94°C for 45 s, 51°C for 30 s, and 72°C for 45 s, and a final extension at 72°C for 7 min. RT-PCR products were analyzed by electrophoresis on a 1% agarose gel stained with ethidium bromide.

### Statistical Analysis

Correlation of the proteome data with previous transcriptome data was analyzed by Pearson correlation using the SPSS software (version 22). GO and pathway enriched analyses were using Hypergeometric distribution method. A *P* < 0.05 was considered significant.

## Results

### Spectrum Data Statistics

**FIGURE 1:**
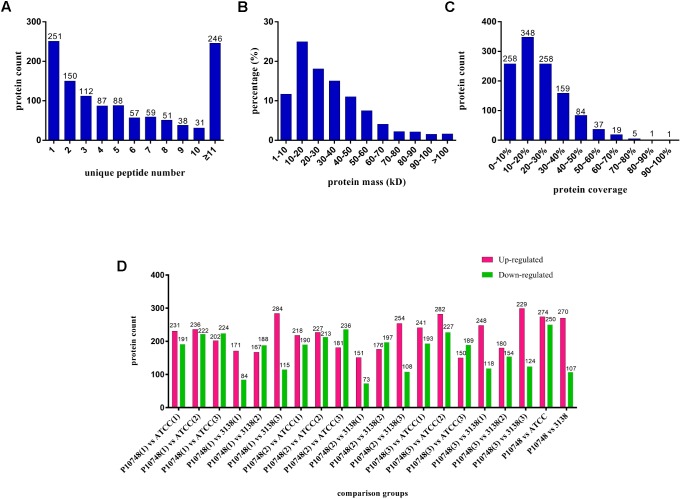
Profiles of membrane proteins identified in three *Enterococcus faecalis* isolates. **(A)** Number of unique peptides identified for individual proteins. *X* axis represents the number of unique peptide. *Y* axis represents the number of proteins. **(B)** Distribution of molecular mass among all identified proteins. *X* axis represents molecular mass (kDa). *Y* axis represents the percentage of the number of proteins. **(C)** Coverage of identified proteins. *X* axis represents coverage, which refers to the proportion of the amino acid sequence of each protein covered by peptides. *Y* axis represents the number of proteins. **(D)** Differentially expressed proteins of each compared group. *X* axis represents each compared group. *Y* axis represents the number of proteins. Up-regulated proteins are indicated in red and down-regulated proteins are indicated in green. Each isolate was tested in triplicate (indicated as 1, 2, and 3 in parentheses) and compared to other isolate separately.

The overall workflow is illustrated in **Supplementary Figure S1**. Nano LC–MS/MS analysis of membrane proteins from three *E. faecalis* isolates P10748, 3138, and ATCC 29212 generated a total of 362,473 raw spectra. With the FDR threshold set to 1%, we obtained 50,635 qualified spectra including 50,285 unique spectra, which were associated with 8,197 peptides (8,144 unique peptides). These peptides were mapped to 1,170 proteins with at least one unique peptide per protein; there were at least two unique peptides for 919 (78.6%) of the 1,170 proteins identified (**Figure 1A**). The majority (92.5%) of these proteins had molecular mass <70 kDa while 7.5% of them were larger than 70 kDa (**Figure 1B**). Most (78%) of these identified proteins had a peptide coverage of >10% while 22% of them had a peptide coverage of <10% (**Figure 1C**).

### Protein Function Prediction

All protein sequences identified were searched against SwissProt, NCBI non-redundant (Nr), TrEMBL, GO, COG, and KEGG databases, resulting in variable rates of matches from 9.66% (113/1,170) with GO to 99.83% (1,168/1,170) with Nr and TrEMBL (**Table 1**). Only one protein (ID: CL14.Contig1_All) didn’t match any sequence in these databases. Based on sequence homology, there were 9, 50, and 151 proteins categorized as surface-, membrane-, and transportation-related proteins, respectively (**Supplementary Table S1**).

**Table 1:** Statistics of protein annotation results.

Database	Nr	SwissProt	TrEMBL	GO	COG	KEGG	All
No. of proteins	1,168	891	1,168	113	1,050	912	1,170

**FIGURE 2:**
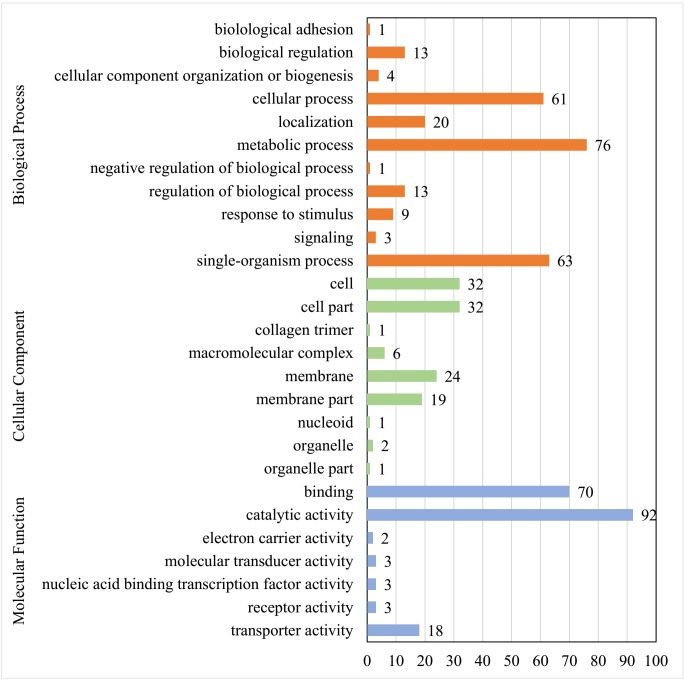
GO function analysis of membrane proteins identified in *E. faecalis*. Proteins were identified by GO terms based on cellular component, molecular function and biological process. *X* axis represents the number of proteins. *Y* axis represents the GO terms.

GO analysis of 113 matched proteins resulted in three broad categories (molecular function, biological process, and cellular component) and 27 GO terms (**Figure 2**). The most abundant GO terms included the catalytic activity (92 proteins) in the molecular function category, the metabolic process (76 proteins) in the biological process category, and the cell (32 proteins), cell part (32 proteins), membrane (24 proteins), and membrane part (19 proteins) in the cellular component category (**Supplementary Table S2**).

**FIGURE 3:**
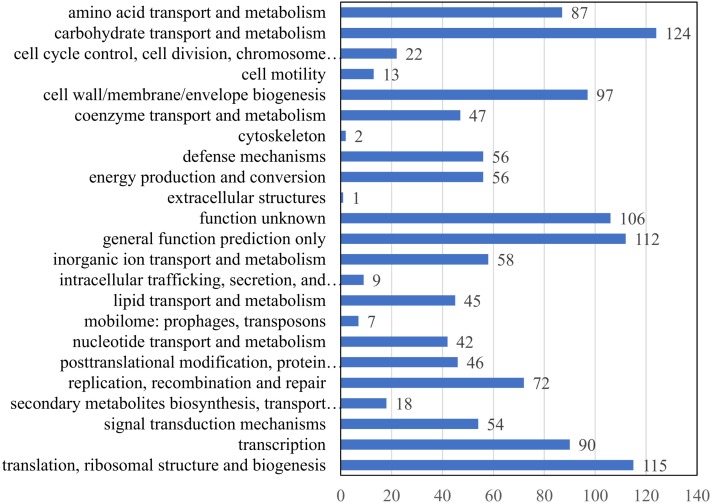
COG function analysis of membrane proteins identified in *E. faecalis*. *X* axis represents the number of proteins. *Y* axis represents the COG terms.

COG analysis of 1,050 matched proteins resulted in 23 COG functional categories (one protein could be assigned to more than one category) as shown in **Figure 3**. The top five abundant categories were carbohydrate transport and metabolism (124 proteins); translation, ribosomal structure, and biogenesis (115 proteins); general function prediction only category (112 proteins); function unknown category (106 proteins); and cell wall/membrane/envelope biogenesis category (97 proteins) (**Supplementary Table S3**).

Based on KEGG analysis, 912 proteins were mapped to 202 pathways (**Supplementary Table S4**). The top five pathways were metabolic (ko01100, 236 proteins, 25.88%), biosynthesis of secondary metabolites (ko01110, 99 proteins, 10.86%), biosynthesis of antibiotics (ko01130, 78 proteins, 8.55%), microbial metabolism in diverse environments (ko01120, 75 proteins, 8.22%), and ABC transporters (ko02010, 52 proteins, 5.70%).

### Identification of Differentially Expressed Proteins Between the Low-Level Linezolid-Resistant and Linezolid-Susceptible Strains

**FIGURE 4:**
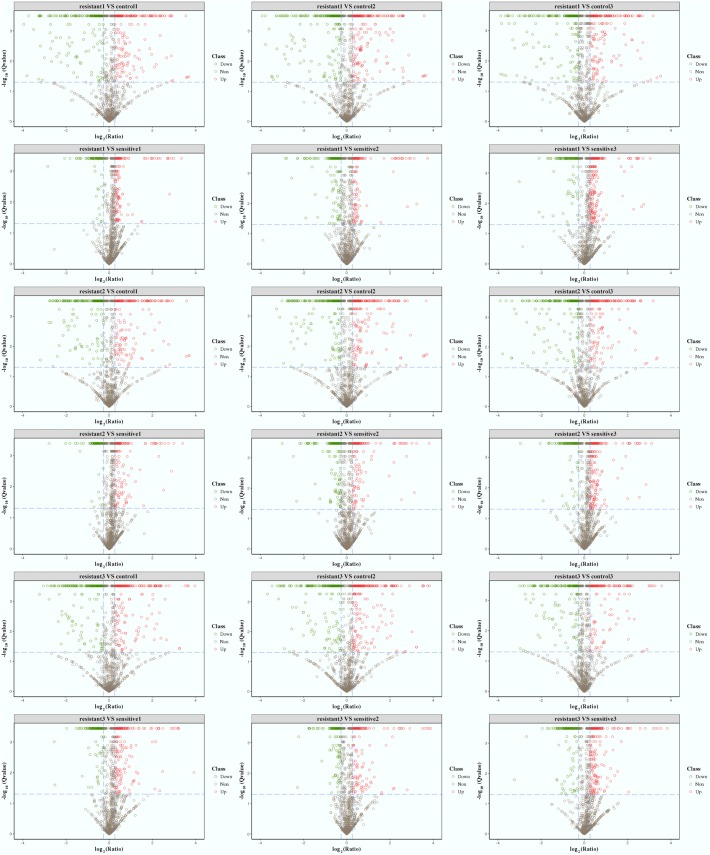
Volcano plots of differentially expressed proteins among the linezolid-resistant strain P10748 (resistant) and two linezolid-susceptible strains 3138 (sensitive) and ATCC 29212 (control). These plots show the confidence and fold change of quantified proteins from each compared group (control1, labeled by tag 126; control 2, labeled by tag 127N; control 3, labeled by tag 127C; resistant1, labeled by tag 129N; resistant2, labeled by tag 128N; resistant3, labeled by tag 128C; sensitive1, labeled by tag 129C; sensitive2, labeled by tag 130C; sensitive3, labeled by tag 130N). Spots with color are highly confident (*Q*-value < 0.05) and differentially expressed proteins. Protein fold changes are expressed as the ratio of all significantly matched peptides. The dashed line represents the applied threshold (*P* ≤ 0.05, Fold change ≥ 1.2). Up-regulated proteins are highlighted in red and down-regulated proteins are highlighted in green. Each isolate was tested in triplicate (indicated as 1, 2, and 3) and compared to other isolate separately.

Based on quantitative spectrum data, significantly differentially expressed proteins between each compared group were identified with a fold change of >1.2 and a *P*-value < 0.05 (**Figures 1D**, **4**). Between the low-level resistant strain P10748 and the susceptible strain ATCC 29212, there were a total of 524 differentially expressed proteins including 274 up-regulated and 250 down-regulated proteins. Between the low-level resistant strain P10748 and the susceptible strain 3138, there were a total of 377 differentially expressed proteins including 270 up-regulated and 107 down-regulated proteins.

**FIGURE 5:**
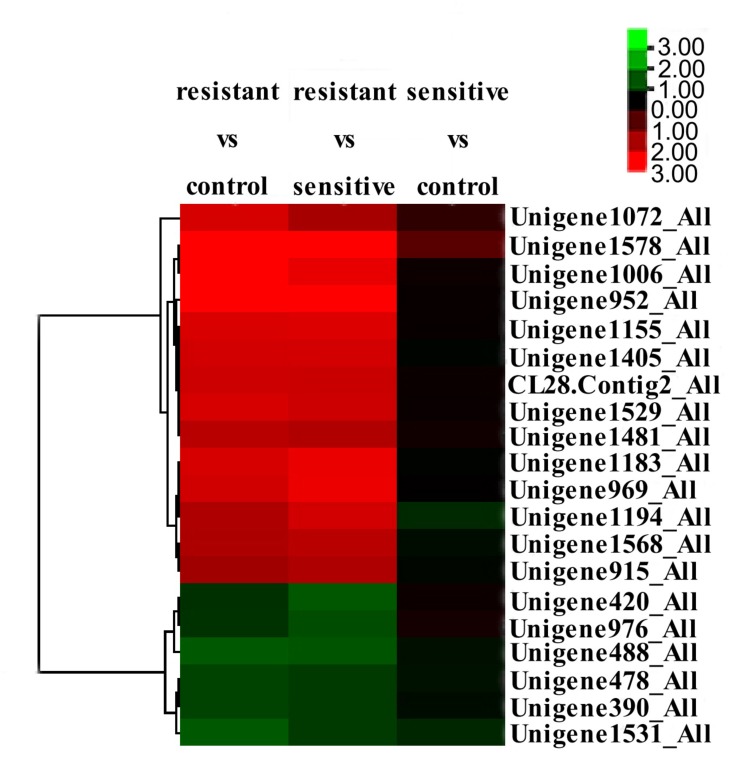
Clustering and heatmap of significantly differentially expressed proteins among the linezolid-resistant strain P10748 (resistant) and two linezolid-susceptible strains 3138 (sensitive) and ATCC 29212 (control). The heat map was created based on the fold changes of abundance of 20 differentially expressed proteins indicated on the right (from Unigene1072_All to Unigene1531_All). The color-coded *z*-score on the up right corner indicates the significance level of protein expression, with red indicating up-regulation and green indicating down regulation.

Combination of the results from these two comparisons led to identification of 14 significantly up-regulated and 6 significantly down-regulated proteins in the low-level resistant strain P10748 compared to the two susceptible strains 3138 and ATCC 29212 (**Table 2**). **Figure 5** shows a heat map of the relative abundance of these differentially expressed proteins. Of note, two significantly up-regulated proteins CL28.Contig2_All and Unigene915_All corresponded to the *esp* and *optrA* genes, respectively, both of which were also significantly up-regulated in our previous transcriptome analysis (Hua et al., 2018). While the surface exclusion protein (Sea1) was significantly up-regulated in proteomics analysis, its transcript was not significantly differentially transcribed in our previous transcriptome analysis (Hua et al., 2018). FexA protein was not identified in proteomics analysis though its transcript was significantly up-regulated in our previous transcriptome analysis (Hua et al., 2018).

**Table 2:** Significantly differentially expressed proteins between a low-level linezolid-resistance strain P10748 and two linezolid-susceptible strains 3138 and ATCC 29212.

Regulation	Protein ID	Mean ratio^∗^ P10748/ATCC 29212	Mean ratio^∗^ 3138/ATCC 29212	Mean ratio^∗^ P10748/3138	Function annotation based on NCBI Nr
Up	Unigene1578_All	28.42	2.05	23.41	Collagen-like protein
Up	Unigene952_All	10.84	1.06	10.35	Alkaline shock response membrane anchor protein AmaP
Up	Unigene1006_All	8.56	1.11	6.66	Surface exclusion protein Sea1
Up	Unigene1155_All	5.94	1.06	6.09	Hypothetical protein, function unknown
Up	Unigene1183_All	5.79	0.97	6.9	DNA-binding protein, XRE family transcriptional regulator
Up	Unigene1529_All	5.78	1.08	5.29	Replication-associated protein RepA
Up	Unigene1072_All	5.75	1.46	3.91	Lipoprotein
Up	Unigene1405_All	5.56	0.96	5.62	Conjugal transfer protein TraB
Up	Unigene969_All	5.53	1.02	6.91	DUF3329 domain-containing protein
Up	CL28.Contig2_All	5.32	1.1	5.2	Enterococcal surface protein (Esp)
Up	Unigene1481_All	4.52	1.14	4.23	Conserved domain protein
Up	Unigene1194_All	4.13	0.72	5.55	Hypothetical protein, function unknown
Up	Unigene1568_All	4.1	0.9	4.58	Hypothetical protein, function unknown
Up	Unigene915_All	3.68	0.95	4.16	ATP-binding cassette protein OptrA
Down	Unigene420_All	0.67	1.11	0.49	Potassium channel protein
Down	Unigene976_All	0.66	1.18	0.53	Conserved hypothetical protein, function unknown
Down	Unigene478_All	0.58	0.87	0.62	Cell division protein FtsK
Down	Unigene390_All	0.58	0.91	0.62	Hypothetical protein, function unknown
Down	Unigene488_All	0.48	0.88	0.5	PTS cellobiose transporter subunit IIC
Down	Unigene1531_All	0.45	0.68	0.64	DNA-binding protein

^∗^*Fold change*.

### Correlation of Proteome Data With Previous Transcriptome Data

Pearson correlation was used to assess the correlation of the significantly differentially expressed proteins identified in this study with their transcription levels reported previously from the same set of *E. faecalis* isolates (Hua et al., 2018). A significant correlation between the protein and mRNA expressions was observed between strains P10748 and ATCC 29212 (Pearson correlation coefficient = 0.675, *P* = 0.001) and between strains P10748 and 3138 (Pearson correlation coefficient = 0.698, *P* = 0.001) (**Table 3**).

**Table 3:** Correlation of significantly differentially expressed proteins with their transcription levels^∗^.

Protein ID	Protein log_2_ FoldChange (P10748/ATCC 29212)	Protein log_2_ FoldChange (P10748/3138)	Transcript ID	mRNA log_2_ FoldChange (P10748/ATCC 29212)	mRNA log_2_ FoldChange (P10748/3138)
Unigene1578_All	4.83	4.55	Unigene1578_All	0	0
Unigene952_All	10.84	10.35	Unigene952_All	9.15	9.15
Unigene1006_All	8.56	6.66	Unigene1006_All	7.38	7.38
Unigene1155_All	2.57	2.6	Unigene1155_All	0	0
Unigene1183_All	5.79	6.9	Unigene1183_All	8.01	8.01
Unigene1529_All	2.53	2.4	Unigene1529_All	0	0
Unigene1072_All	2.52	1.97	Unigene1072_All	0	0
Unigene1405_All	5.56	5.62	Unigene1405_All	6.29	6.29
Unigene969_All	2.47	2.79	Unigene969_All	0	0
CL28.Contig2_All	5.32	5.2	CL28.Contig2_All	14.11	14.11
Unigene1481_All	2.18	2.08	Unigene1481_All	0	0
Unigene1194_All	4.13	5.55	Unigene1194_All	9.23	9.23
Unigene1568_All	4.1	4.58	Unigene1568_All	4.39	4.39
Unigene915_All	3.68	4.16	Unigene915_All	11.83	14.42
Unigene420_All	-0.58	-1.02	Unigene420_All	0	0
Unigene976_All	0.66	0.53	Unigene976_All	1.1	0.98
Unigene478_All	-0.79	-0.69	Unigene478_All	0	0
Unigene390_All	0.58	0.62	Unigene390_All	-5.73	-5.73
Unigene488_All	-1.01	-1	Unigene488_All	0	0
Unigene1531_All	-1.15	-0.64	Unigene1531_All	0	0

^∗^*Based on RNA-Seq data (Hua et al., 2018). Zero indicates undetectable transcripts for both strains. Pearson correlation coefficient of protein and mRNA expressions between strains P10748 and ATCC 29212 is 0.675, *P* = 0.001. Pearson correlation coefficient of protein and mRNA expressions between strains P10748 and 3138 is 0.698, *P* = 0.001*.

### GO and Pathway Enrichment Analysis

In GO enrichment analysis, between the resistant strain P10748 and susceptible strain ATCC 29212 there were 11, 13, and 41 enriched GO-terms in the cellular component, molecular function, and the biology process categories, respectively; between the resistant strain P10748 and susceptible strain 3138, there were 11, 10, and 12 enriched GO-terms in the cellular component, molecular function, and the biology process categories, respectively (**Supplementary Table S5**). The most enriched GO terms for differentially expressed proteins in the resistant strain P10748 compared to the susceptible strain ATCC 29212 included the primary active transmembrane transporter activity in the molecular function category, mental ion transport in the biology process category, and membrane in the cellular component category. The most enriched GO terms for differentially expressed proteins in the resistant strain P10748 compared to the susceptible strain 3138 included the substrate-specific transmembrane transporter activity in the molecular function category, ion transport in the biology process category, and membrane in the cellular component category.

Enriched pathways for differentially expressed proteins in each compared group were diverse from each other, and mainly associated with quorum sensing (QS), phosphotransferase system (PTS), and pantothenate and CoA biosynthesis pathways (**Supplementary Table S5**).

### Verification of *sea1* Gene and Its Transcription

Since the transcript of *sea1* was not detected in our previous transcriptome analysis (Hua et al., 2018), we wanted to rule out the possibility that the high-level Sea1 protein expression revealed by proteomics analysis of the low-level linezolid-resistant strain P10748 resulted from an artifact. When PCR was used to amplify the *sea1* gene in strain P10748 along with strains 3138 and ATCC 29212 strains, a strong, single band with an expected size was present in strain P10748 but absent in other two strains (Data not shown). Based on sequence analysis of the PCR product, the nucleotide sequence of the *sea1* gene in strain P10748 was 91% identical to the *E. faecalis* plasmid pAD1 (GenBank No. X62658.1) (Weidlich et al., 1992) and 98% identical to the *E. faecalis* plasmid pMG2200 (GenBank No. AB374546.1) (Zheng et al., 2009). This finding confirms the presence of *sea1* gene in strain P10748.

To verify the transcription level of the *sea1* gene in different *E. faecalis* isolates, we performed RT-PCR using total RNA and found that this gene was highly transcribed in strain P10748 while no transcription was detected in strains 3138 or ATCC 29212 strains (**Supplementary Figure S2**).

## Discussion

To understand the role of membrane proteins in low-level linezolid-resistant *E. faecalis*, we performed a quantitative proteomics analysis of membrane proteins in one low-level linezolid-resistant strain P10748 and two linezolid-susceptible strains 3138 and ATCC 29212, all of which have been previously studied by whole transcriptome analysis (Hua et al., 2018). We identified a total of 14 significantly up-regulated and 6 significantly down-regulated proteins in strain P10748 compared to strains 3138 and ATCC 29212 (**Figure 5** and **Table 2**), which were in general positively correlated with transcription levels revealed in previous transcriptome analysis (**Table 3**). Included among the most significantly up-regulated proteins were OptrA, Esp, and Sea1. We speculate that these three proteins are the main determinants of low-level linezolid-resistance in *E. faecalis.*

OptrA protein may play a major role in mediating low-level linezolid-resistance in *E. faecalis.* The knowledge of the mechanism by which the OptrA protein mediates antibiotic resistance has evolved substantially over time. The *optrA* gene was originally identified in 2012 as an ABC transporter from the *E. faecalis* strain 599 (GenBank Accession No. EJU90935), which represents a part of the reference genome for the Human Microbiome Project (Human Microbiome Jumpstart Reference Strains Consortium Nelson et al., 2010). Subsequently, this gene was found to be prevalent in *E. faecalis* as well as *E. faecium* in China as a transferable element in plasmid or chromosomal DNA that can confer oxazolidinone and phenicol transferable resistance (so designated as *optrA*) (Cai et al., 2015; Wang et al., 2015).

Recently, this gene has also been detected in clinical isolates of *Enterococcus* spp. from North America and Europe (Brenciani et al., 2016; Flamm et al., 2016; Cavaco et al., 2017; Gawryszewska et al., 2017; Pfaller et al., 2017), as well as in staphylococci (Li et al., 2016; Butin et al., 2017). Phylogenetic analysis has suggested that OptrA belongs to the ABC-F protein subfamily, which is widespread in Gram-positive bacteria (Murina et al., 2018). The vast majority of earlier studies have postulated that the ABC-F proteins mediate antibiotic resistance by acting as an active efflux pump capable of exporting antibiotics out of the bacterial cell (Eckford and Sharom, 2009; Wang et al., 2015; Gawryszewska et al., 2017; Hua et al., 2018). It was not until very recently that direct compelling evidence became available that these proteins act instead to protect the bacterial translational machinery from antibiotic-mediated inhibition by displacing bound antibiotics from the ribosome (Sharkey et al., 2016; Sharkey and O’Neill, 2018). In both this study (**Figure 5** and **Table 2**) and our previous transcriptomics analysis (Hua et al., 2018), OptrA was consistently significantly up-regulated in the low-level linezolid-resistant *E. faecalis* strain P10748, supporting its role in mediating linezolid-resistance.

Of note, *optrA* gene has been commonly detected in *E. faecalis* and *E. faecium* isolates with no or low-level resistance to linezolid (MIC = 2–16 mg/L) (Cai et al., 2015; Wang et al., 2015; Morroni et al., 2017). However, there are only a very few studies reporting the transcription or translation levels of this gene in clinical isolates (Brenciani et al., 2016; Hua et al., 2018). In our previous quantitative RT-PCR (qRT-PCR) study of three strains with low-level linezolid-resistance (including strain P10748, MIC = 8–16 mg/L) and two strains with high level linezolid-resistance (MIC = 192–256 mg/L) and 23S rRNA mutations, we found a significantly higher level of *optrA* transcription in the former strains than in latter strains (Hua et al., 2018). This observation raises the possibility of a generally higher expression of *optrA* in low-level linezolid-resistant strains than high level linezolid-resistant strains with 23S rRNA mutations. This possibility awaits confirmation in the future using a large number of samples.

While *optrA* has been well-recognized as a transferable resistance gene, the mechanism and regulation of *optrA* transfer remain poorly understood. The results of the present study shed some light on this question. First of all, the significantly up-regulated expression of the Sea1 protein (**Figure 5** and **Table 2**) in the linezolid-resistant strain P10748 implies a potential role in regulating resistance gene transfer. This protein is known to be located on the surface of donor bacterial cells, with a primary function of reducing the frequency of futile conjugation of sex pheromone plasmids between donor strains (Weidlich et al., 1992). In this study, we detected a significantly up-regulated expression of the Sea1 protein in proteomics analysis as well as a strong transcription in RT-PCR strain P10748 (**Supplementary Figure S2**). Currently, it is unknown if the *optrA* gene is carried on a sex pheromone plasmid in strain P10748 in our studies and there has been no report of any *E. faecalis* isolates carrying *optrA* on sex pheromone plasmids. In this regard, we have initiated an effort to determine the complete chromosome and plasmid sequences in multiple *optrA*-positive *E. faecalis* isolates, which will be published separately.

In addition to Sea1 protein, a number of other proteins in strain P10748 were also significantly up-regulated that are likely involved in controlling conjugative plasmid transfer (**Figure 5** and **Table 2**). The conjugal transfer protein TraB (Unigene1405_All) may serve as an inhibitor to suppress self-induction (Bazan et al., 2013). The XRE family transcription regulator protein (Unigene1183_All) may control the expression of plasmid genes (Do and Kumaraswami, 2016). The replication-associated protein RepA (Unigene1529_All) belongs to a family of initiator proteins encoded by several low-copy plasmids from *Enterococcus*, *Staphylococcus*, *Lactobacillus*, *Lactococcus*, and *Bacillus* species and is likely involved in controlling the replication of plasmid DNA (Chattoraj et al., 1988; Francia et al., 2004). The RepA identified in our study is 100% identical to the RepA protein identified in *E. faecalis* plasmids pKUB3006 and pKUB3007 (GenBank Nos. AP018539.1 and AP018544.1), and 95% identical to the RepA protein in *E. faecalis* plasmid Efsorialis-p1 (GenBank No. CP015884.1). The dramatic simultaneous up-regulation of all these proteins suggests that conjugative activity is highly active in the linezolid-resistant strain, which may facilitate the spread of linezolid resistant genes like *OptrA*. Clearly, further studies are needed to elucidate whether these proteins interact each other or with additional molecules and how they regulate the conjugative plasmid transfer.

Our proteomics analysis of the linezolid-resistant strain P10748 also revealed four significantly up-regulated proteins that are likely involved in biofilm formation, including the Esp (CL28.Contig2_All), collagen-like protein (Unigene1578_All), alkaline shock response membrane anchor protein AmaP (Unigene952_All), and lipoprotein (Unigene1072_All). Among these four proteins, only Esp has been directly linked to biofilm formation in *E. faecalis*; the remaining three have only been shown to be involved in biofilm formation in other bacteria, such as collagen-like protein in *Streptococcus* spp. (Tsatsaronis et al., 2013; Lukomski et al., 2017), AmaP in *S. aureus* (Muller et al., 2014), and lipoprotein in *Actinobacillus pleuropneumoniae* (Xie et al., 2016). Esp protein is encoded on a pathogenicity island (Huycke et al., 1991), which is highly prevalent in *E. faecalis* and *E. faecium* isolates with resistance to linezolid, vancomycin, ampicillin, tetracycline, and chloramphenicol (Willems et al., 2001; Kafil and Mobarez, 2015; Zheng et al., 2017). Considerable studies have demonstrated the involvement of Esp in biofilm formation in *E. faecalis* (Toledo-Arana et al., 2001; Tendolkar et al., 2004; Tian et al., 2014; Zheng et al., 2017). In both this proteomics analysis (**Figure 5** and **Table 2**) and our previous transcriptomics analysis (Hua et al., 2018), Esp was consistently significantly up-regulated in the low-level linezolid-resistant *E. faecalis* strain P10748, supporting the involvement of Esp in mediating linezolid-resistance through biofilm formation. The possibility of this mechanism is further supported by the significant enrichment in strain P10748 of cell structure and biological pathways associated with biofilm formation from this study and previous study (Hua et al., 2018), including the cell wall/membrane/envelope biogenesis, carbohydrate metabolism, and QS pathway. Biofilm formation can contribute to antibiotic resistance through multiple actions such as reduced antibiotic penetration, nutrient limitation and slow growth, adaptive stress responses, and formation of persister cells (Stewart, 2002). In addition, biofilm formation can enhance conjugation of drug-resistance plasmids (Stalder and Top, 2016). Nonetheless, the role of Esp in biofilm formation is not supported by some studies (Creti et al., 2004; Kristich et al., 2004). It has been generally accepted that Esp is not compulsorily required for biofilm formation but its presence may enhance biofilm formation (Mohamed et al., 2004).

This study has the following main limitations. First, only one *E. faecalis* strain with low-level linezolid-resistance and two linezolid-susceptible strains of *E. faecalis* were analyzed, and it is uncertain if the results can be generalized. Clearly, further studies are needed of additional strains with similar levels of linezolid-resistance or susceptibility. Second, this study did not include strains with a high-level, clinically relevant resistance. It is unknown if the significantly differentially expressed proteins in the low-level linezolid-resistant strain are also expressed in similar levels in strains with clinically relevant resistance, and thus not unique for low-level linezolid-resistant strains.

## Conclusion

This report represents the first investigation of the mechanisms of drug resistance in *E. faecalis* by a quantitative proteomics approach. Our analysis suggests that the low-level linezolid-resistance in *E. faecalis* is conferred primarily by the ABC protein OptrA through ribosomal protection and, possibly, also by the Esp and other proteins through biofilm formation. The genetic transfer of *optrA* is potentially regulated by the surface exclusion protein Sea1, conjugal transfer protein TraB, replication protein RepA and XRE family transcription regulator protein. This study not only shows the power of quantitative proteomics in cell membrane analysis but also provides a foundation for further in-depth studies on the mechanisms of antibiotic resistance in *E. faecalis.*

## Author Contributions

YX conceived the study and designed experiments, with assistance of LM. JY performed all experiments except TMT labeling and nano-LC–MS/MS. MY, JZ, YC, and DZ assisted in experiments. All authors contributed to data analysis, manuscript drafting, revising, and approved the manuscript submission.

## Conflict of Interest Statement

The authors declare that the research was conducted in the absence of any commercial or financial relationships that could be construed as a potential conflict of interest.

## Supplementary Material

FIGURE S1 Experimental workflow of quantitative proteomics analysis of membrane proteins in *Enterococcus faecalis*.

FIGURE S2 Agarose gel electrophoresis analysis of reverse-transcription PCR products of the *sea1* gene from three *E. faecalis* isolates. Lane M, DNA size marker; lane 1, strain P10748; lane 2, strain ATCC 29212; lane 3, strain 3138. The gel was stained with ethidium bromide. A fragment of 698 bp was present only in strain P10748 and absent in other two strains.

TABLE S1 Total information of all identified proteins.

TABLE S2 Information of GO analysis.

TABLE S3 Information of COG analysis.

TABLE S4 Information of pathway analysis.

TABLE S5 Information of enriched GO and pathway analysis.
