# Supplementary figures and images for: Quantitative Proteomics Analysis of Membrane Proteins in Enterococcus faecalis With Low-Level Linezolid-Resistance
Source: Front Microbiol. 2018 Jul 27;9:1698. doi: 10.3389/fmicb.2018.01698 (PMC6072972; doi:10.3389/fmicb.2018.01698)

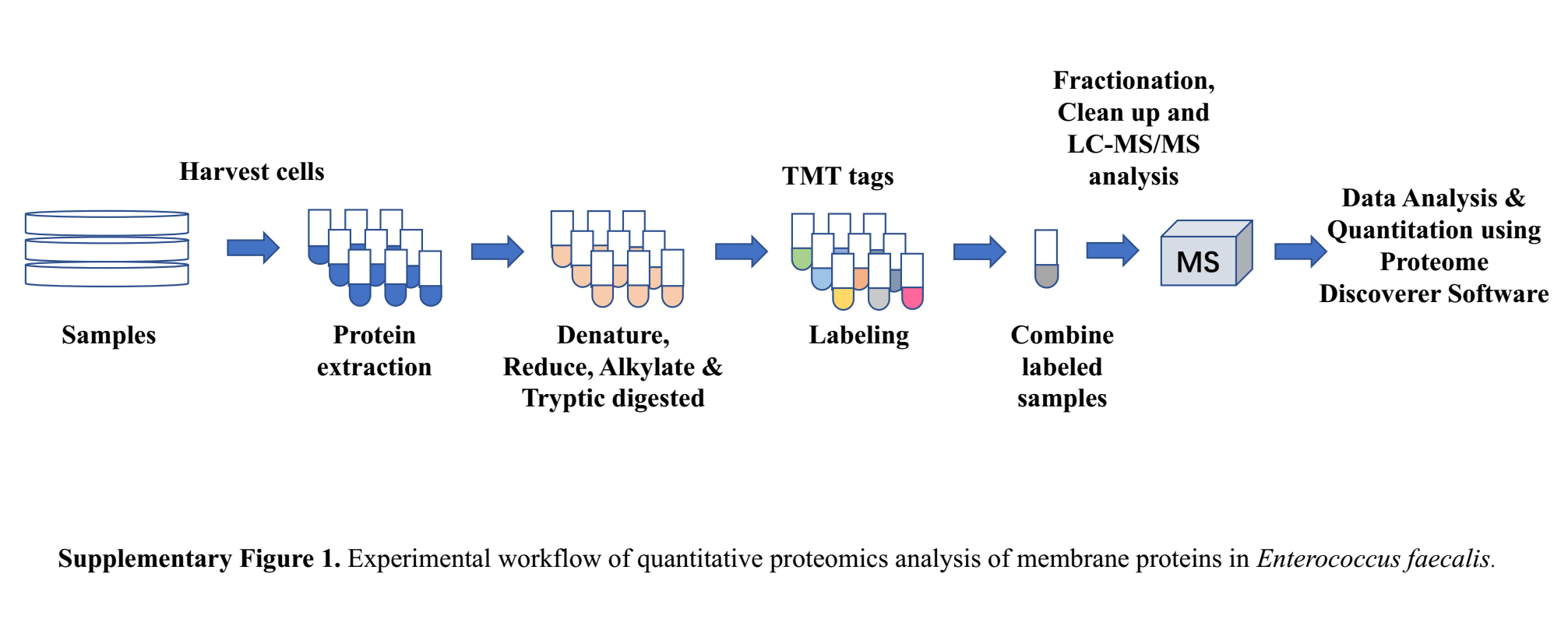

Supplement: FIGURE S1 — Experimental workflow of quantitative proteomics analysis of membrane proteins in Enterococcus faecalis. [file Image_1.png]

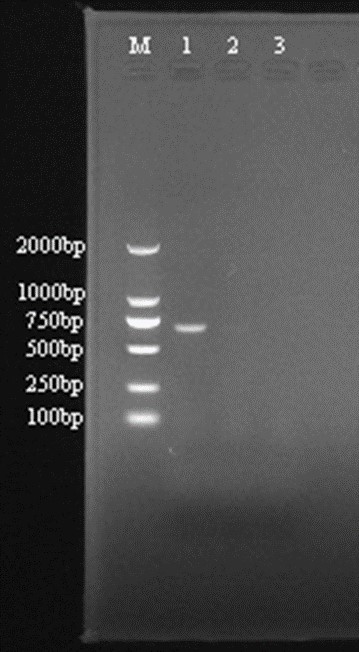

Supplement: FIGURE S2 — Agarose gel electrophoresis analysis of reverse-transcription PCR products of the sea1 gene from three E. faecalis isolates. Lane M, DNA size marker; lane 1, strain P10748; lane 2, strain ATCC 29212; lane 3, strain 3138. The gel was stained with ethidium bromide. A fragment of 698 bp was present only in strain P10748 and absent in other two strains. [file Image_2.JPEG]
